# Sleep deprivation and corneal chronobiology: reevaluating overnight corneal changes

**DOI:** 10.1038/s41598-024-84431-y

**Published:** 2025-01-04

**Authors:** Zsuzsa Zakarné Aszalós, Bence Lajos Kolozsvári, Vivien Lénárt, Dorottya Pásztor, Ziad Hassan, Éva Surányi, Reda Chaker, Mariann Fodor

**Affiliations:** 1 Department of Ophthalmology, Faculty of Medicine, University of Debrecen, Nagyerdei blvd. 98, Debrecen, 4012 Hungary; 2 Orbident Refractive Surgery and Medical Center, Nagyerdei krt. 98, Debrecen, 4012 Hungary

**Keywords:** Cornea, Circadian changes, Sleep deprivation, Anterior segment of the eye, Scheimpflug parameters, Pachymetry, Keratometry, Physiology, Medical research

## Abstract

This prospective cohort study is aimed to investigate circadian variations in corneal parameters, focusing on sleep-deprived subjects. Sixty-four healthy individuals (age range: 21–76 years) actively participated in this study, undergoing examinations at least five times within a 24-hour timeframe. The analysis encompassed keratometric parameters of the cornea’s front (F) and back (B) surfaces, refractive power in flattest and steepest axes (K1, K2), astigmatism (Astig) and its axis (Axis), aspheric coefficient (Asph), corneal pachymetry values of thinnest corneal thickness (Pachy Min) and corneal thickness in the center of the pupil (Pachy Pupil), volume relative to the 3 and 10 mm corneal diagonal (Vol D3, Vol D10) and surface variance index (ISV). Circadian changes were assessed using a hierarchical, mixed-effects linear regression adjusted for age and night shift. A total of 1,636 measurements revealed significant circadian changes in various corneal parameters, including K1 F, K2 F/B, Astig F/B, Asph F/B, Pachy Min/Pupil, Vol D3/10, and ISV (*p* < 0.0001). Moreover, K1 B exhibited a significant circadian change (*p* = 0.0002), while Axis F/B remained unchanged. Notably, Corneal thickness peaked before 6 o’clock in the morning and reached its minimum after 12 o’clock. Contrary to previous notions linking corneal diurnal changes with eyelid closure during sleep, our study reveals that these changes persist in the absence of sleep. This research contributes valuable insights into the impact of sleep deprivation on corneal properties, warranting further investigations to deepen our understanding of daily variations in visual quality and guide the planning of refractive eye surgery interventions.

## Introduction

The cornea undergoes continuous environmental interactions. Its refractive power, curvature, thickness, and transparency are essential for proper vision, forming clear images on the retina. Modern tools offer precise corneal parameter insights, crucial for understanding its anatomy and physiology^1,2^.

The body’s physiological systems follow a 24-hour cycle (circadian rhythm), regulated by the organism’s endogenous clock. The diurnal rhythm is controlled by the day-night cycles. Vision, intricately linked to light intensity changes throughout the day, operates rhythmically^3^. Corneal influences, such as age, gender, ethnicity, estrogen, cortisol and melatonin levels, fasting and external temperatures, remain to be elucidated. Within the context of the whole eye, the retina plays a crucial role in corneal phase resetting^3^. Understanding daily avascular corneal changes, an important part of vision, is vital for clinical and scientific purposes. It aids in diagnosing systemic disorders, corneal diseases like ectasias, monitoring dystrophies, pre and post-operative assessments for refractive eye surgeries. These insights also impact myopia progression and retinal detachment^1,4,5^.

Several studies utilizing various tools have explored diurnal changes in the human cornea. Irrespective of the instruments employed, research consistently reveals that the cornea is thickest upon waking up in the morning^6–8^. Conversely, it attains its thinnest point between 4 and 12 h after waking^9–17^. Corneal curvature also exhibits diurnal variation: it is flattest upon waking up and becomes steeper at the anterior surface in the early evening. Spherical equivalent values indicate hyperopia in the morning, shifting slightly myopic throughout the day^1,15,17^. Fluctuations in spherical aberration, corneal sensitivity, tear pH, tear film stability and volume, and tear film osmolarity have been studied throughout the day^1,10,18,19^. Additionally, the diurnal rhythm also impacts eye pressure, axial length, and retinal and choroidal thickness^6,7,9,10,16,20,21^.

In scientific literature, the effects of sleep deprivation on corneal properties is an overlooked area of research, despite the widespread prevalence of sleep disorders due to modern lifestyle factors^22^. To the best of our knowledge, there has not been a study addressing the circadian variation of corneal parameters during sleep deprivation, particularly regarding corneal volume and the posterior surface. Our study aims to fill this gap by investigating circadian changes in the cornea of healthy eyes, employing a large participant pool and utilizing Pentacam, known for its excellent repeatability^2^.

## Methods

### Subjects and procedure

Our prospective observational study involved 64 healthy participants of European descent with specific inclusion criteria, including 20/20 Snellen equivalent distance visual acuity, lower refraction error (< 3.0 diopters [D]), normal tear meniscus, and an absence of ophthalmological disorders. Participants, primarily students and employees, provided written consent in accordance with the Helsinki guidelines, and the study protocol received approval from the University of Debrecen’s Research Ethics Committee (DE RKEB/IKEB: 5418 − 2020). Participants fell under either of two-groups, those of “night-shift working nurses” and those who are not “non-nurses” (e.g., students, ophthalmologists). To represent the real world 12 nurses were involved in our study accounting for 18.75% of the 64 participants. Exclusion criteria encompassed systemic or local drug treatments, use of eyedrops, refraction errors exceeding 3.0 D (pathologic myopia and hyperopia), inflammatory or infective systemic or ocular diseases, contact lens usage, dry eye, prior ocular surgeries, abnormalities in the lens or retina, chemical injuries, delayed epithelial healing, age below 18 years, and pregnancy or lactation. Furthermore, the participants had no sleep disorders, used no sleep medicine, and did not travel across time zones within one month before the examinations. Before slit lamp examinations (to exclude any ophthalmological abnormalities), each eye underwent three scans with Pentacam (Pentacam AXL, Oculus Optikgeräte GmbH, Wetzlar, Germany, software version 1.25r15). The rotating Scheimpflug camera captured 25 images/second in auto mode at perfect eye-set with repeats for disturbances like blinking or image distortion. Data from three sessions were averaged into session-level values for analysis.

Each participant underwent measurements every 3–4 h within a 24-hour period, with at least 5 measurements a day. The study offered two measurement options for the subjects: 1, six measurements within a single 24-hour interval without sleep (scheduled between 05:30 − 08:30, 09:00–11:30, 12:30 − 15:00, 16:00–18:30, 20:00–23:30, 00:30 − 04:30); or 2, mostly with nurses, 5 measurements within each of two 12-hour intervals not more than one week apart. In detail, during the “morning shift” these were around 06:30, 09:00, 12:30, 15:00, and 17:30. During the “night shift” (these are the sleep-deprived subjects), the measurements were around 18:00, 21:00, 00:00, 03:00 and 05:30. The nurses regularly worked in two shifts and regularly spent a third of their nights awake and working. Other subjects never worked in shifts and slept regularly at night, except when nocturnal measurements were taken for the study. Enthusiastic participants partook in both options to ensure comprehensive 24-hour coverage. Participants engaged in regular activities between measurements, avoiding alcohol and drug consumption. The sleep-deprived subjects were not in dark or low-blue-light environments, nor were their eyes closed. The participants were not allowed to sleep between night measurements. This period was spent either working at the study site or participating in work meetings. Additionally, some participants went home to check on their family members and then immediately returned to the test site.

Keratometric parameters of the cornea’s front (F) and back (B) surfaces, including refractive power measure along the flattest and steepest axes (K1, K2), astigmatism (Astig) and its axis (Axis), and aspheric coefficient (Asph Q) relative to the 8 mm corneal diagonal, were measured. Corneal pachymetry values, such as thinnest corneal thickness and corneal thickness at the pupil center (Pachy Min, Pachy Pupil), along with volume relative to the 3 mm and 10 mm corneal diagonals (Vol D3, Vol D10), were recorded. The surface variance index (ISV), a corneal-specific index, was calculated and exported to Microsoft Excel (Microsoft Corp, Redmond, Washington) for subsequent statistical analysis.

### Statistical analysis

One eye per participant was randomly selected for assessment, and multiple measurement sessions within the same subject were assigned a session identifier. For descriptive purposes, the outcome variables’ observed limits (minimum and maximum) were utilized to derive the observed ranges of within-subject circadian change. Minimum, maximum, and range values were described across subjects employing standard statistical methods.

Circadian variation was evaluated through regression modeling using trigonometric predictors based on sine and cosine of multiples of 2π, 4π, and 6π derived from exam time as fractional time of day. These predictors, adjusted for age and subject group (night-shift working nurses, versus non-nurses), were fixed-effect terms in multilevel mixed-effects linear regression with a random intercept allowed to vary independently across subjects and sessions. Circadian variations were assessed in terms of minimum and maximum outcome predicted by the fixed-effects component of the model for a non-nurse subject of sample mean age, times of day when these extremes were observed, and estimated difference between modeled maximum and minimum expressed as a point estimate with p-value and 95% confidence interval. Scatter plots illustrating trends were created by plotting fitted outcome values against time of day, removing subject- and session-level variability and subject-group effects (these are referred to as ‘shifted’ fitted values). The tendency at the sample mean age was superimposed. Statistical significance was attributed to p-values < 0.05, indicating a significant difference.

## Results

Our study included sixty-four randomly selected eyes of 64 healthy volunteers of European descent (37 women and 27 men). The mean age was 32.3 years (SD: 12.3, range: 20.6–76.2 years). A total of 1,636 measurements were performed.

### Circadian changes in keratometry values including astigmatism and its axis

The flat- (K1) and the steep-axis (K2) keratometry values, corneal astigmatism (Astig) and its axis of both (the front (F) and the back (B)) surface, were analyzed. Table 1 shows the minimum and maximum average values, the range of the parameters for keratometry values as well as the amount and time points of the minimum and maximum values. Except for the axis of astigmatism on both surfaces, all parameters show a significant circadian change (all *p* < 0.0001; except K1B *p* = 0.0002). While the keratometry values (K1, K2) on the front side of the cornea reached the minimum values in the early morning hours (between 05:12 − 05:15), and the highest values around 10:19 − 10:24 in the morning. The changes on the back surface did not occur at the same time as the posterior surface values, their lowest level also in the early morning (between 05:09 − 06:21), however, they reached their maximum value between 17:56 − 18:32. The astigmatism of the front surface reaches its minimum value at 17:18 and its maximum value at 11:27, however, all these occur at 21:41 and 03:53 on the back surface, respectively. Neither the front surface nor the back surface astigmatism axis changes during the day diurnally (*p* = 0.39 and 0.42).

**Table 1:** Circadian changes of keratometry parameters in healthy subjects (*N* = 64) measured with Pentacam AXL (K1 = flat-axis keratometric value in diopters (D) on anterior (F) and posterior (B) corneal surface; K2 = steep-axis keratometric value in diopters (D) on anterior (F) and posterior (B) corneal surface; astig = corneal astigmatism of the front (F) and the back (B) surface; SD = standard deviation; p = p-value). *Significant difference.

	Minimum (mean, SD; min-max)	Maximum (mean, SD; min-max)	Range (mean, SD; min-max)	Value and time of minimum	Value and time of maximum	Significance value of the circadian change
K1 F	42.63, 1.48; 37.6–45.9	42.96, 1.5; 37.9–46.2	0.33, 0.18; 0.1–1.2	42.61, 05:12	42.68, 10:24	*p* < 0.0001^*^
K2 F	43.55, 1.47; 39.1–46.2	43.97, 1.5; 39.5–47.2	0.42, 0.18; 0.2–1.1	43.56, 05:15	43.63, 10:19	*p* < 0.0001^*^
K1 B	− 6.21, 0.23; − 6.7 to − 5.8	− 6.12, 0.22; − 6.7 to − 5.7	0.091, 0.05; 0–0.2	− 6.154, 06:21	− 6.136, 17:56	*p* = 0.0002^*^
K2 B	− 6.52, 0.25; − 7.3 to − 6.1	− 6.41, 0.25; − 7.1 to − 6.0	0.12, 0.06; 0–0.3	− 6.473, 05:09	− 6.446, 18:32	*p* < 0.0001^*^
Astig F	0.66, 0.63; − 1.8–1.6	1.16, 0.42; 0.3–1.9	0.498, 0.63; 0.1–3.7	0.84, 17:18	0.95, 11:27	*p* < 0.0001^*^
Astig B	0.25, 0.12; 0–0.5	0.37, 0.12; 0.1–0.6	0.11, 0.06; 0–0.3	0.30, 21:41	0.32, 03:53	*p* < 0.0001^*^

**Fig. 1:**
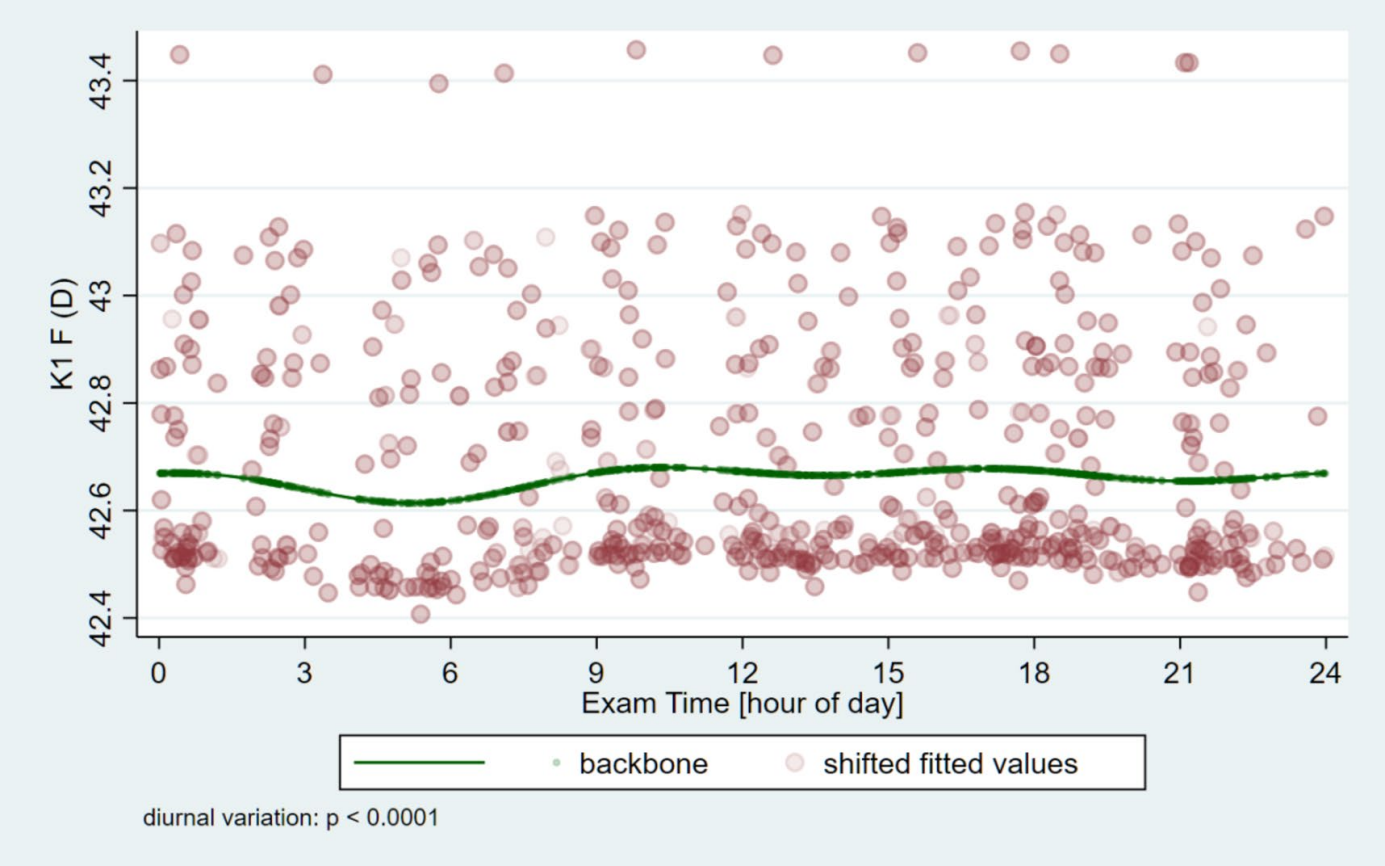
Circadian change of the flat-keratometry (K1) on the front surface of the cornea. The lowest value is reached early in the morning (at 05:12 AM) and the highest value is reached also in the morning (10:24 AM), after that a relatively constant value is seen throughout the day.

**Fig. 2:**
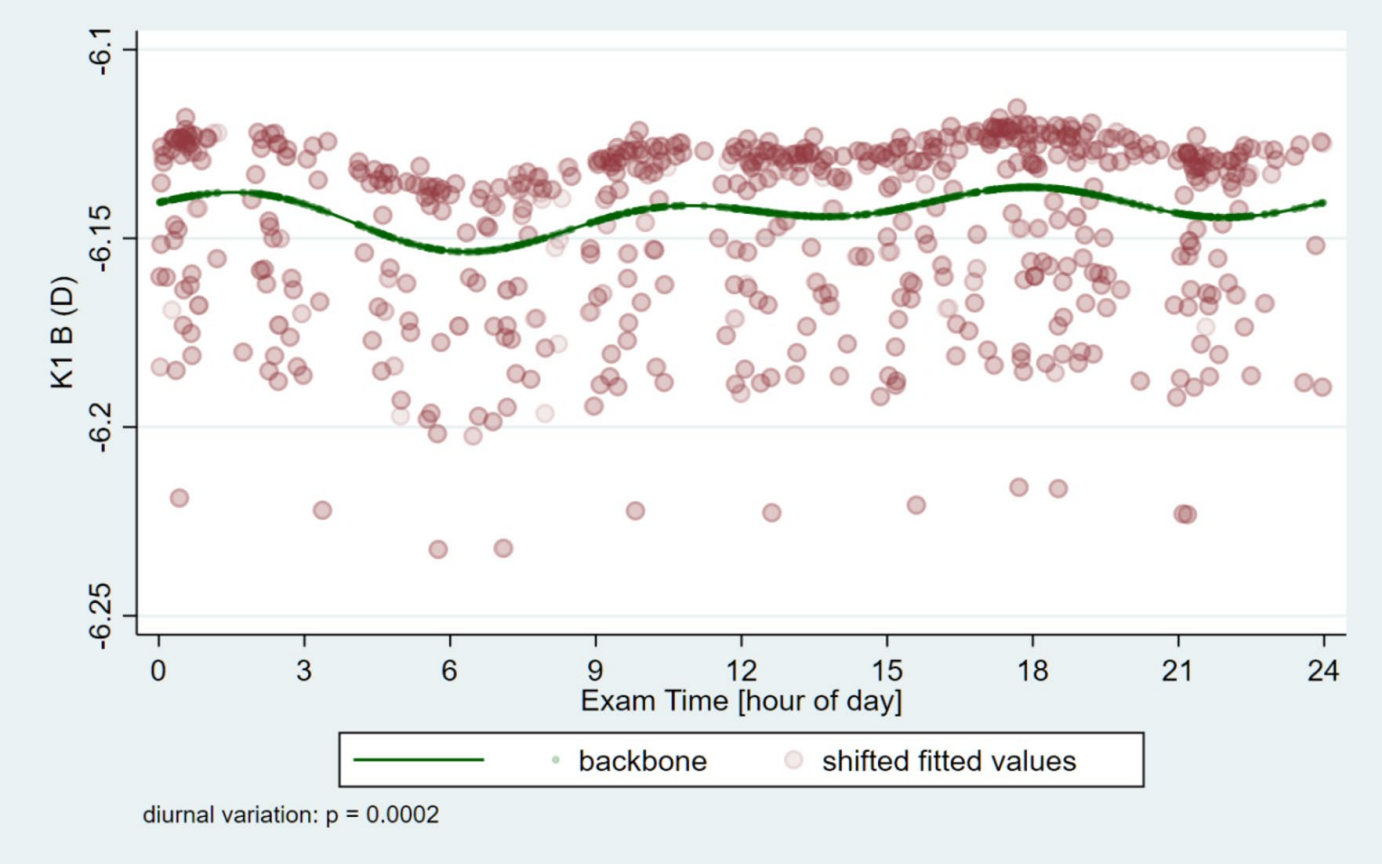
Circadian change of the flat-keratometry (K1) on the back surface of the cornea. The lowest value was measured in the early morning hours (06:21 AM), then it fluctuated several times during the day, while the highest value was reached in the afternoon (15:56 PM).

**Fig. 3:**
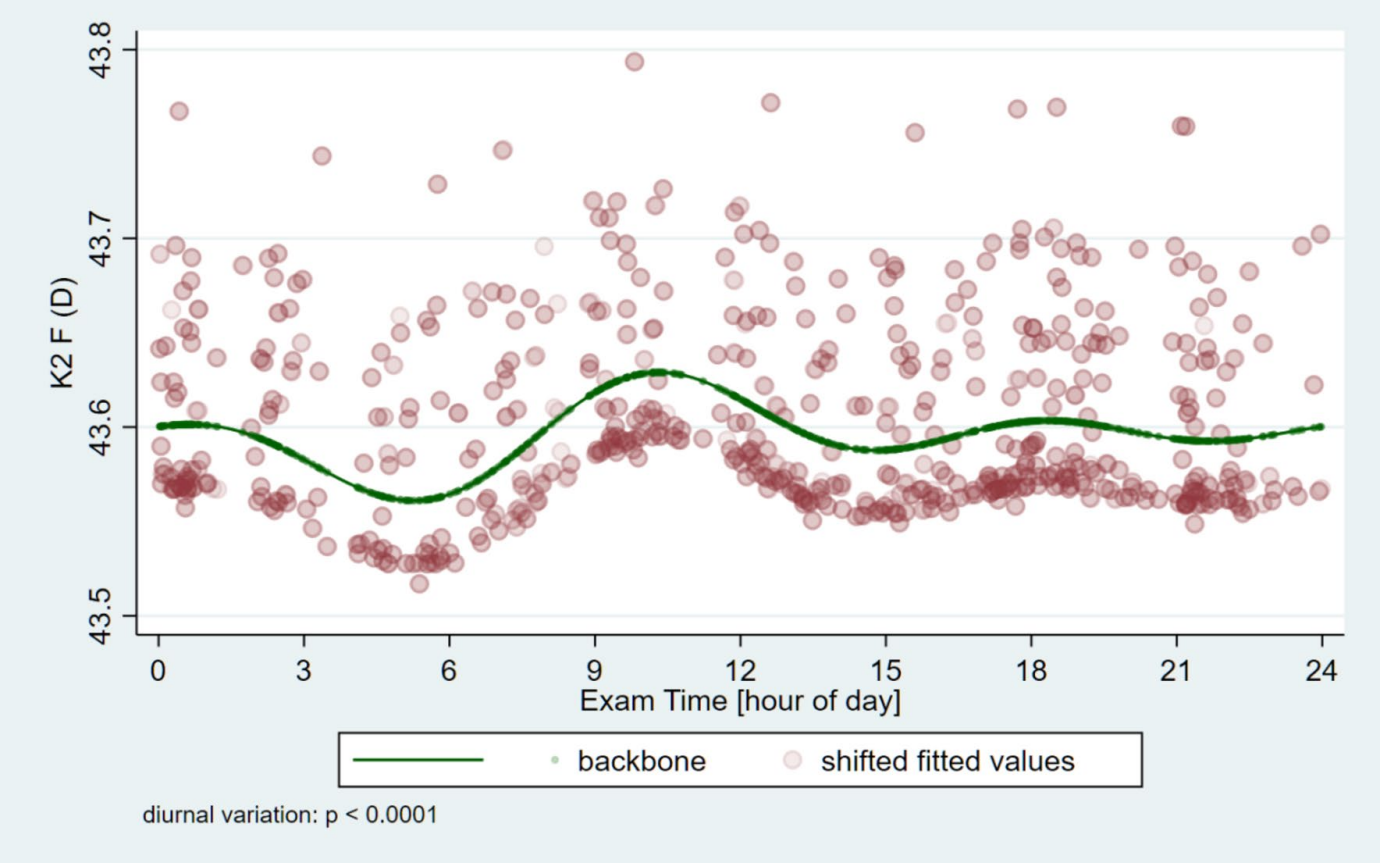
Circadian change of the steep-keratometry (K2) on the front surface of the cornea. It shows its lowest value in the early morning hours (05:15 AM), after which it rises until it reaches its highest value in the morning hours (10:19 AM), after which it starts to decrease.

**Fig. 4:**
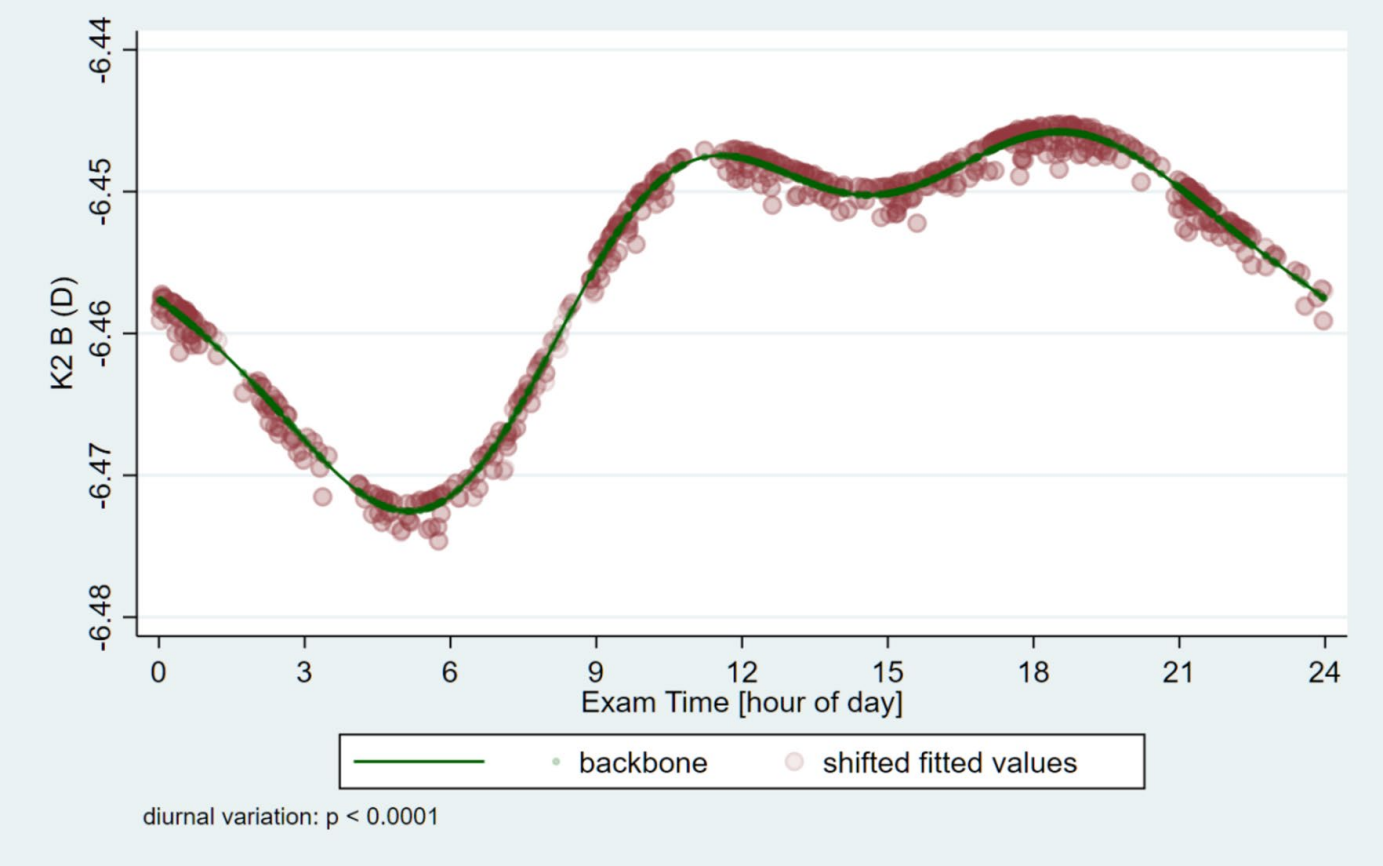
Circadian change of the flat-keratometry (K2) on the back surface of the cornea. It shows its lowest value in the early morning hours, then we see a sawtooth rise until it reaches its maximum during the evenings.

The circadian change curves obtained by modeling estimation are shown in Figs. 1, 2, 3 and 4.

### Circadian changes of corneal pachymetry values and ISV

**Fig. 5:**
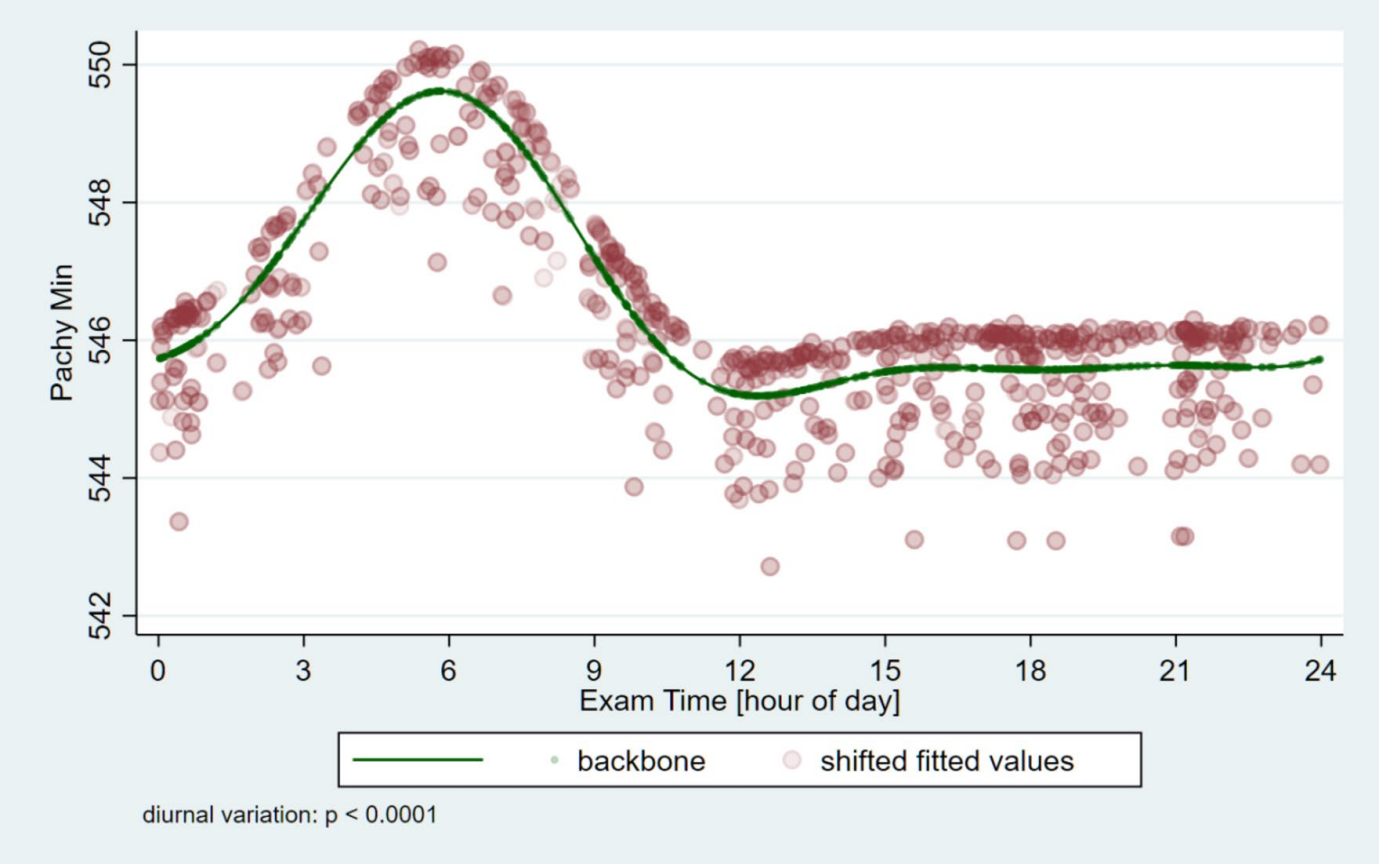
Circadian change of the corneal thickness at the thinnest point of the cornea (Pachy Min). The highest value is reached early in the morning, and then after a sudden decrease, a relatively constant value throughout the day is seen.

**Fig. 6:**
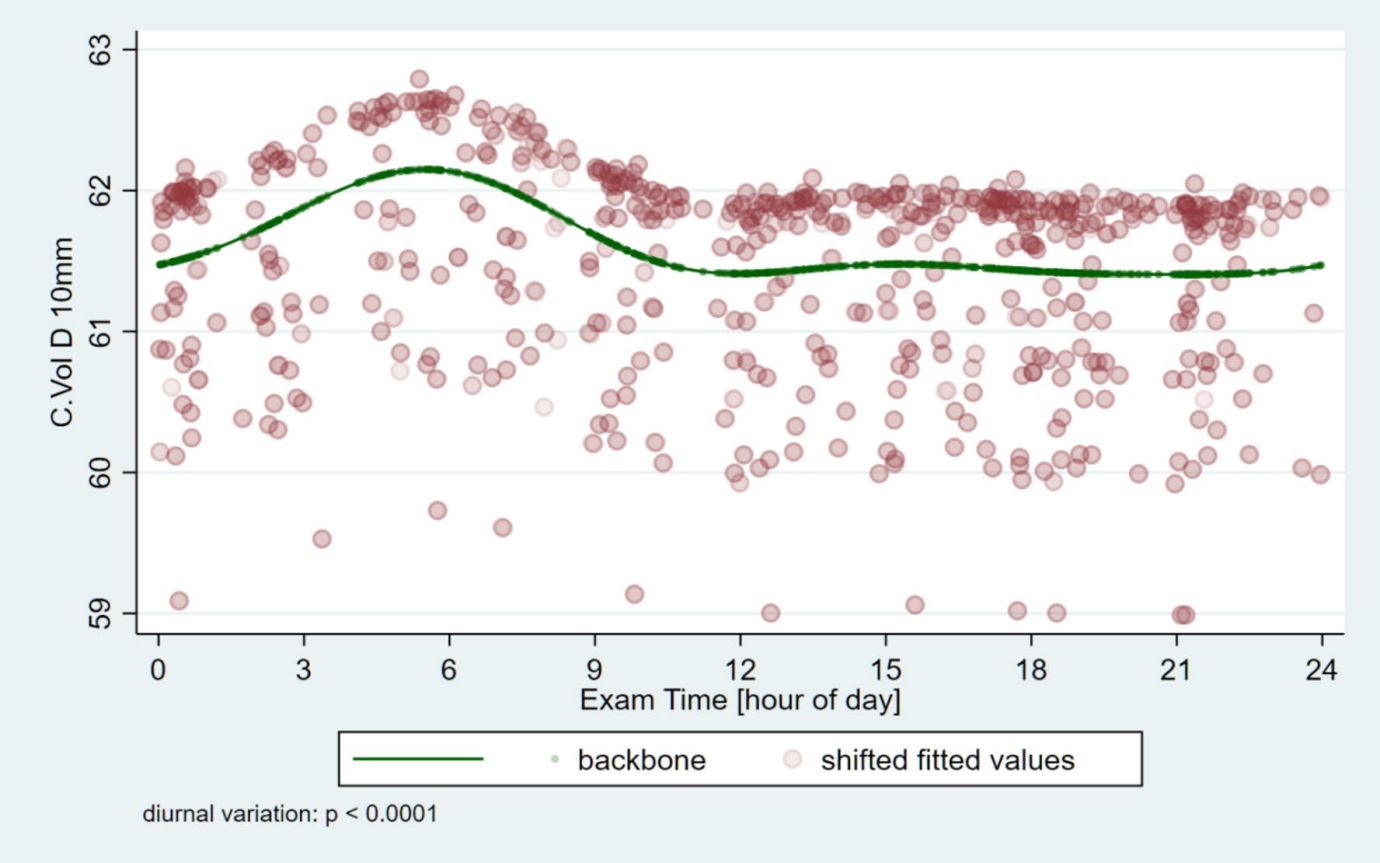
Circadian variation of the corneal volume relative to a 10 mm diagonal (Vol D10). It reaches its highest value in the early morning hours, decreases during the day, reaches its lowest value during the evening, and then increases again.

**Fig. 7:**
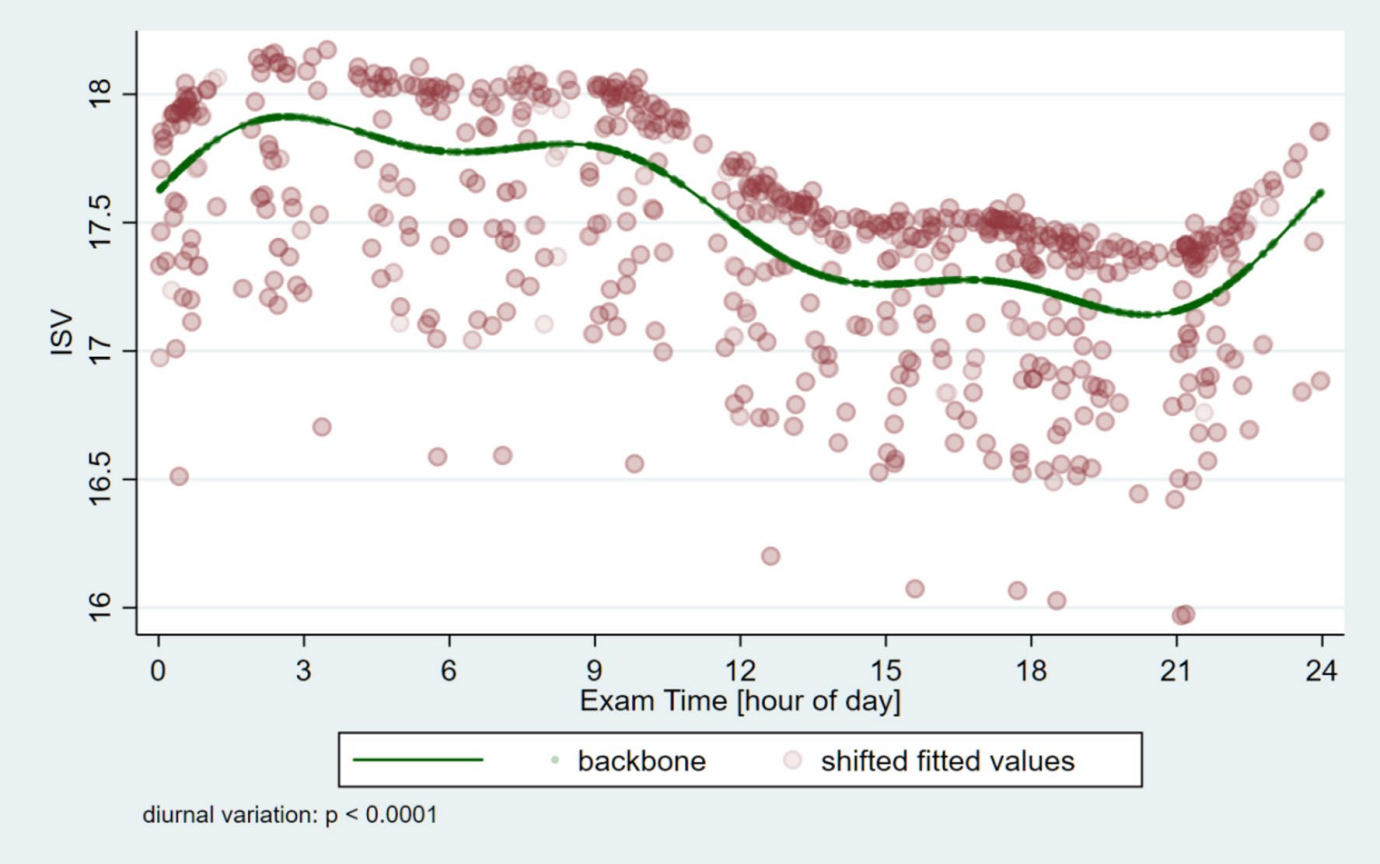
Circadian variation of the index of corneal surface variance (ISV). It reaches its highest value in the morning hours, while after a continuous decrease, it reaches its lowest value in the evening hours.

The corneal thickness at the thinnest point of the cornea (Pachy Min) and at the pupil’s center (Pachy Pupil) as well as the volume of the cornea in a diameter of 3 mm and 10 mm centered on the anterior corneal apex (Vol D 3 and 10 mm) were analyzed. Table 2 shows the minimum and maximum average values and the variable of the parameters for the pachymetric and ISV data as well as the amount and time points of the minimum and maximum values. For all four pachymetric parameters, and also for ISV the significance of circadian change is *p* < 0.0001 (Figs. 5, 6 and 7). The thickness values of the cornea reach the early morning hours maximum values (around 05:31 − 05:49 AM), while the lowest values are around 12:23 − 12:26, except Vol D10 which reaches its maximum value eight and a half hours later (at 20:23 PM).

**Table 2:** Circadian changes of pachymetric and volumetric corneal parameters and index of surface variation in healthy subjects (*N* = 64) measured with Pentacam AXL (Pachy Min: corneal thickness at the thinnest point of the cornea (µm); Pachy Pupil: corneal thickness at the pupil’s center (µm); vol D3 mm and vol D10 mm: volume of the cornea in a diameter of 3 mm and 10 mm, centered on the anterior corneal apex (mm^3^), ISV = index of surface variation; SD = standard deviation; p = p-value) *Significant difference.

	Minimum (mean, SD; min-max)	Maximum (mean, SD; min-max)	Range (mean, SD; min-max)	value and time of minimum	value and time of maximum	Significance value of the circadian change
Pachy Min	539.3, 32.9; 457–633	553.8, 34.2; 462–648	14.5, 6.2; 2–41	545.2, 12:23	549.6, 05:49	*p* < 0.0001^*^
Pachy Pupil	542.6, 33.0; 459–634	556.6, 34.2; 466–649	14.0, 5.8; 3–38	548.3, 12:26	552.7, 05:56	*p* < 0.0001^*^
Vol D3	3.94, 0.24; 3.4–4.6	4.04, 0.25; 3.4–4.7	0.097, 0.059; 0-0.3	3.97, 12:25	4.01, 05:51	*p* < 0.0001^*^
Vol D10	60.8, 3.3; 54.6–69.8	62.7, 3.5; 56.5–72.6	1.87, 0.75; 0.8–4.3	61.4, 21:01	62.2, 05:31	*p* < 0.0001^*^
ISV	15.3, 5.5; 7–43	19.4, 6.5; 9–48	4.1, 2.8; 1–15	17.1, 20:23	17.9, 02:39	*p* < 0.0001^*^

The ISV (index of surface variation) characterizes the irregularity of the surface, showing significant circadian variation (Table 2; Fig. 7). The modeling estimates of the circadian change can be characterized by the following data: the minimum value of ISV is 17.1, which can be seen on the curve at 20:23 PM, while its maximum value is 17.9 at 02:39 AM (*p* < 0.0001).

### Circadian changes of corneal asphericity

**Fig. 8:**
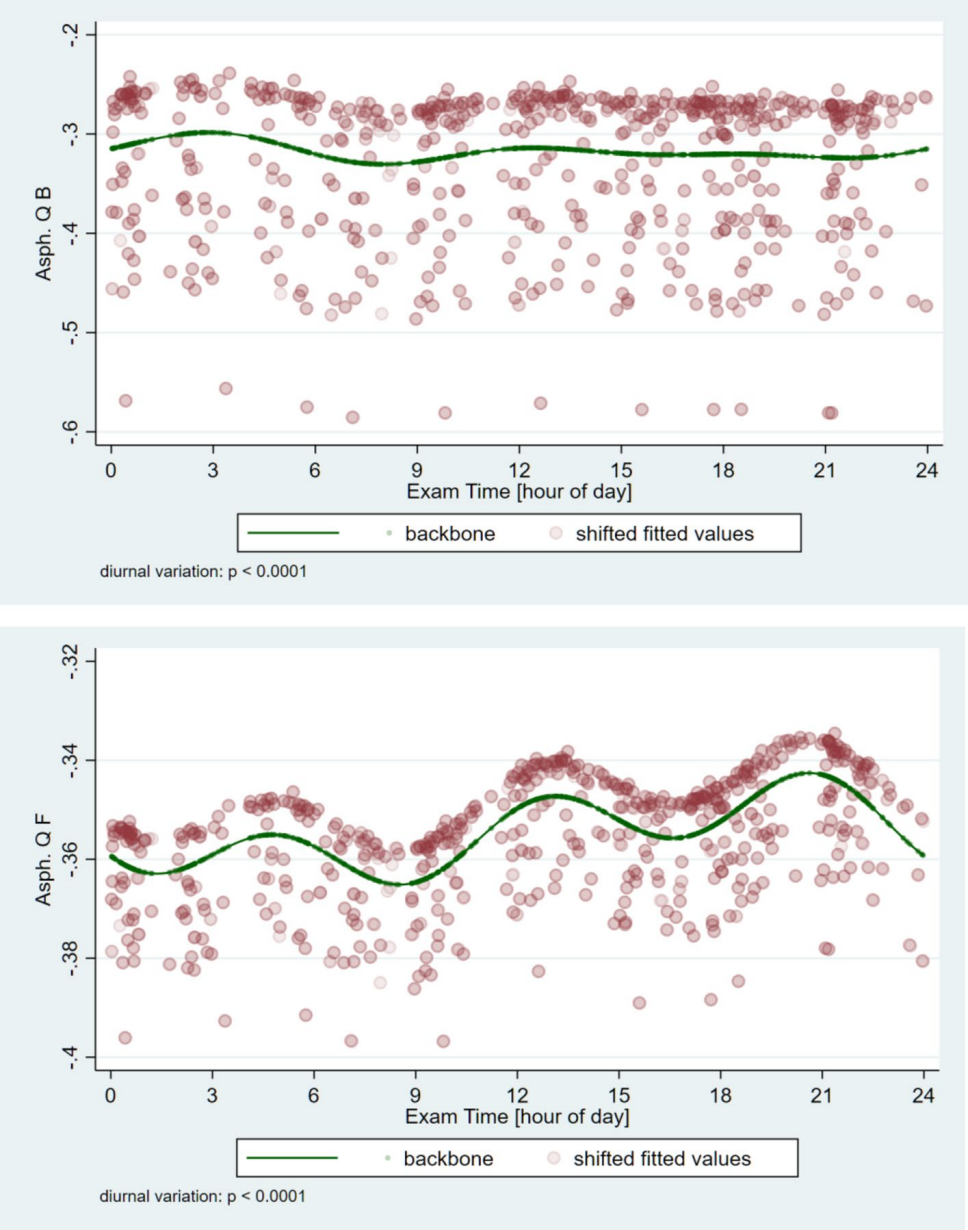
Circadian variations of the aspheric coefficient (Asph.) of the front (F) and back (B) surfaces of the cornea. Significant and continuous fluctuations throughout the day can be detected.

The minimum average value of the aspheric coefficient on the front side of the cornea (Asph. Q F) is -0.42 (SD: 0.22, min.-max.: -1.06- 0.76) and the maximum average is -0.28 (SD: 0.2, min.-max.: -0.79- 0.84). Asph. Q F reaches the minimum value (-0.37) in the morning (08:31 AM1), and the highest value (-0.34) at 20:37 PM in the evening, showing a significant circadian variation (*p* < 0.0001). For the back surface, the minimum mean value is -0.37 (SD: 0.22, min.-max.: -1.1- 0.0) and the maximum mean value is -0.21 (SD: 0.18, min.-max.: -0.73-0.12). The minimum value of Asph. Q B is -0.33, which can be seen on the curve at 08:00 AM, while its maximum value of -0.30 can be seen at 02:52 AM (*p* < 0.0001) (Fig. 8).

## Discussion

Our study analyzes the circadian variations of the cornea, responsible for the two-thirds of the eye’s refractive power, across a 24-hour measurement period. This analysis incorporates participants across a wide age range and introduces new standards in sample size and measurement sessions. While the inclusion of a diverse age range is notable, it’s crucial to acknowledge that sleep patterns vary by age, and thus, further analysis of age-related differences within sub-groups is warranted. Our research presents significant information about the alterations, including the anterior and posterior corneal surfaces with multiple measurements revealing the true within-subject changes, highlighting significant circadian changes in the examined corneal parameters.

The corneal pachymetric nature serves as an indicator of the cornea’s metabolic status, offering insights into corneal hydration^13^. Consistent with previous findings in the literature, our results indicate changes of approximately 1% in corneal curvature and 3% in pachymetric values, with the most notable changes in keratometric and pachymetric values occurring early in the morning^5–7,9–15,23^. Previous studies, despite variations in instruments, duration, and measurement timings, consistently showed that the cornea is thickest right after eye-opening in the morning and thinnest after 5 to 10 h after waking^10,13,15^. Our study is the first to measure human corneal thickness through the night without sleeping and the data is based on real measurements and not extrapolated results. To our surprise, the thickness of the cornea increased during the nocturnal period in sleep-deprived participants, as if they had slept. This contrasts with previous assumptions; however, those studies either did not take measurements at night or only included participants waking up from sleep^5–7,9–13,15,16,18,19,24,25^. In our study, all measurements taken between 03:00 AM and 06:00 AM were conducted on sleep-deprived subjects. During the early nocturnal hours, there was a rapid increase in pachymetry data, and the cornea reached its peak thickness early in the morning (around 05:31 − 05:36), despite eyelids remaining open and the presence of artificial lighting. The estimated difference between the modeled thinnest corneal thickness (PachyMin) at its minimum and maximum was 4.424 μm, a significant circadian change (*p* < 0.0001). Interestingly the mean minimum in central corneal thickness occurred at 22:30 p.m., just before the subjects went to sleep in Read’s study^7^, which is in contrast with our study, because the lowest values were around 12:23 − 12:26. Some earlier studies could not find significant pachymetric changes over time, however, these were established either by examining a small number of individuals, or a narrow age group, or carrying out only few measurements during a narrow period of the day^5,8,10,12,13,15,23,26,27^. Additionally, previous studies have typically investigated diurnal changes within a single day and rarely included measurements extending past 9:00 PM., resulting in a time coverage of only 12–15 h^1,12,15,16,27^.

According to earlier studies, the cornea is the thickest during the early morning hours, exhibiting overnight swelling ranging from 3 to 13%. This can partially be attributed to the period of sleep with closed eyelids and can be partly explained by reduced tear production, drainage, evaporation, and osmolality coupled with hypoxia, causing water inflow into the stroma^6,7,11,13,18,19,24,28^. Upon awakening, tear osmolarity nearly stabilizes within a few minutes during the day due to reflex tears induced by eye-opening, serving as a protective mechanism^19^. It has been hypothesized, that tear film tonicity may only be partially responsible for corneal hydration control and has no effect on deswelling state. Instead, the endothelial pump system is believed to be primarily responsible for this process^18^. The corneal epithelium absorbs oxygen from the air, and the recovery of overnight swelling is slower for the cornea than that of the epithelium^11^. Corneal endothelial pump function and tear evaporation are the two major mechanisms for removing water from the corneal stroma and epithelium. Feng and his colleagues hypothesized, that under the closed eyelid, decreased oxygen pressure mainly affects the endothelial pump function and as the eyes open, the evaporation of the tear film quickly increases causing a relatively fast de-swelling of the epithelium^11^. Harper et al. found, that in three out of eight participants after waking up the cornea thickness continued to increase^13^. Burfield et al. examined a relatively large number of healthy young individuals for 24 h which included sleeping in the laboratory. They were confident that the short periods of moderate illumination did not interrupt the diurnal variations. They performed night examinations at 0:00 AM and 4:00 AM with all lights off from 11:00 PM to 7:00 AM. The central corneal thickness was the thickest at 4:00 AM and the thinnest at 8:00 PM^9^. In another study without measurements during the sleeping periods, it was the thickest at 6:00 AM and the thinnest at 22:30 PM^7^.

Based on measurements from only fifteen young volunteers spending one day in a sleep laboratory, corneal thickness was found to be thicker during the nocturnal/sleep period than during the diurnal/wake period^24^. The oscillation of several corneal parameters throughout the day is influenced by internal and external environmental factors such as intraocular pressure, forces exerted by the eyelids on the anterior cornea, blinking frequency, reading, tear production/evaporation, and changes associated with prolonged eye closure, as well as air humidity and temperature^10,17,25^, however, the complex mechanism regulating corneal hydration still has to be further elucidated. Corneal homeostasis is a complex process, involving the barrier function of the limiting layers, the pump function of the endothelium, and osmotic interactions with the tears are crucial components in maintaining corneal thickness and transparency^4,11^.

Utilizing the Pentacam instrument, we observed circadian variations in corneal volume. A similar pattern was observed for the volume of the cornea concerning the 3 mm and 10 mm diagonals but with greater amplitude in the peripheral cornea. The maximum values were reached in the early morning hours. Regarding the volume related to the 3 mm diagonal, within the optical zone crucial for vision, a decrease was observed until it reached its minimum value in the middle of the day. It then stabilized and began increasing during the night with the start of the next cycle. On the other hand, despite the similar pattern, for the larger volume zone with reference to the 10 mm diagonal, it reached its minimum value in the evening at 20:23 PM, which is a unique time among pachymetric values. The non-uniform circadian variation observed in corneal volume from the center to the periphery in our study may be related to the different collagen fiber densities of the cornea between the two zones^28^. Further investigation into corneal hydration dynamics is warranted, as these findings remain unexplained.

The curvature and refractive power of the cornea play an important role in vision and exhibit a diurnal pattern. In previous studies examined only 32 subjects and only twice a day (10:00 AM and 18:00 PM) could detect the steepening of the anterior corneal topographic parameters (K1/K2/Kmax)^1,15,17^. Still, posterior topographic indices remained unchanged during the day^17^. In contrast to these findings, we were able to detect significant circadian variations on the posterior surface of the cornea, although the change in the front surface was greater compared to the back. This can be explained by the environmental influences affecting the front surface, whereas the back surface is in contact with the aqueous humor, experiencing fewer external influences. Studying diurnal corneal variation necessitates highly repeatable methods such as Scheimpflug imaging and a sufficiently large sample size, covering a complete 24-hour period to yield accurate data. In our study, the minimum refractive power values for both the anterior and posterior surfaces were observed in early morning hours when the cornea was at its thickest. When corneal edema occurs, the refractive power of the cornea decreases because part of the tissue volume is replaced by water^11^. Our findings indicate that the maximum refractive power is not reached simultaneously by the anterior and posterior corneal surfaces. The front surface peaks in the morning, whereas the back surface peaks late afternoon and evening. Attention has recently shifted toward the importance of the back surface because it plays an essential role in astigmatism and spherical aberrations of the eye. Furthermore, examination of the posterior corneal surface may also be important for the early detection of keratoconus, and ectasia in the examination of pre-and postoperative refractive surgery patients^5,29^. Studies with limited sample size and lacking nocturnal measurements did not show significant fluctuations in central corneal curvature or meanK throughout the day^14,23^; however, another study revealed no change in central corneal curvature overnight, but decrease in curvature between 7:00 AM and 9:00 AM with significant fluctuation over the 24-hour period were detected^6^.

Astigmatism often results from the non-ideal shape of the cornea, leading to blurred vision. The pressure exerted by the eyelids has been implicated in the etiology of with-the-rule astigmatism. Earlier studies have shown significant diurnal variation in low-order astigmatism^1^. Our results indicate that the degree of astigmatism changes differently during the day on the anterior and posterior surfaces of the cornea. On the front surface, it peaks in the morning, while its minimum value is reached in the late afternoon. Conversely, the posterior surface exhibits an inverse pattern, reaching its lowest value in the evening and peaking early morning hours. Astigmatism of both surfaces showed significant circadian variations. The posterior surface compensates for nearly 31% of the astigmatism of the anterior surface^30^. Despite changes in the degree of astigmatism, its axis does not change significantly on any of the corneal surfaces. The stability of the axis during the day is presumably of paramount importance in order to ensure stable vision. The network sustaining the adaptation of the eye physiology to the 24-hour cycle is complex and has to be further elucidated.

The spherical aberration of the corneal surface is influenced by corneal asphericity, corneal curvature, and entrance pupil diameter influence^31^. Diurnal variation in spherical aberration has been observed^1^. The cornea is an aspherical surface, thicker at the edges than in the middle, working in conjunction with the crystalline lens to optimize focus on the macula^32^. The aspheric coefficient can characterize the degree of asphericity. Although no correlation was found between the asphericity of the front and back surfaces, both surfaces undergo significant changes with age^30^. Our study revealed significant and dynamic circadian fluctuation during the day while Kiely et al., observed fluctuation in the values but constant asphericity throughout the day, using a photokeratoscope^15^. The quality of vision is optimized by the asphericity of the cornea. Slight changes can modulate the focus of peripheral light significantly; thus, its diurnal change plays an important role in visual function. These results may serve as a suitable reference for designing aspheric intraocular lenses and keratorefractive surgery.

In our examination of the corneal topometric index we focused on the surface variation index (ISV) as it characterizes the irregularity of the eyes’ surface, its circadian changes have not been investigated yet. The changes in ISV specifically pertain to the anterior corneal surface and its changes may be related to the changes in the tear film covering the dynamically changing anterior corneal surface. We observed that ISV peaked during the night and early morning hours, and then continuously decreased during the day, it was lower during the daytime hours. Interestingly it reached the lowest value in the evening at 20.23 PM. A lower value is ideal for better vision. Changes in ISV may represent local tear film changes, as precorneal tear film itself undergoes diurnal variation in both composition and stability^33^. The circadian variation in ISV found in this study is in part likely to be another source because it reaches the lowest value in the evening. It may be partially related to pressure from the eyelids leading to the displacement of epithelial tissue^25^. Activities involving close work also plays a role in the diurnal corneal variation and corneal aberrations are the lowest in the early morning (before the subject commences significant close work)^25^.

Based on the number of measurements performed on the largest cohort of patients to date analyzing the circadian corneal variations, to the best of our knowledge, this study offers significant insights of changes in the cornea compared to previous literature. Our study includes the widest age range reported so far in a circadian study of this nature (21 to 76 years). The strength of our study is that among the participants there were night shift workers and subjects who stayed late at night or until morning, whose night measurements reflected less physiological states, but it may aid in the understanding of the “true” values and thus our night data are not extrapolated or missing results as in earlier studies. With this selection of patients, we simulate the real world, since a significant percentage of the population works at night. The main result of our investigation is that similar curves to previous studies with extrapolated data were obtained in our cohort with night shift and overnight subjects, proving that the fluctuation of the corneal parameters was not only caused by night sleep but by complex mechanisms including the alternation of days and nights too.

Limitations of this study include the fact that it was a single-center study including only European descendants without considering the menstrual cycle, plasma level of cortisol, diet, smoking status, caffeine consumption, subjects’ weight, heating system, type and duration of indoor electric lights, outside temperature, air humidity, duration of eyelid closure or sleeping patterns, and previous day’s possible alcohol consumption^34^. However, during the study, the participants worked, studied, and commuted, which did not allow for significant alcohol consumption. The pupil width is measured by the Pentacam, but we did not calculate it separately, although the measurements were made in the same room, with the same machine, in complete darkness. The diurnal rhythm in IOP is mediated via neuronal and hormonal signaling outside the eye^3^, however, we have not currently investigated these aspects. The eye responds to the variations of the surrounding conditions and in this way tear film and its pH, stability, volume, and osmolarity also show diurnal variations^18,19^. Examination of these factors could have provided additional information during our investigation. None of the sleep-deprived subjects were in dark or low-blue-light environments, nor were they permitted to close their eyes, although this could have offered further insights into the influence of different melatonin levels on corneal circadian fluctuations. Finally, systemic and physiologic factors, e.g., state of hydration were also not assessed. The transparency of the cornea resulted from a constant hydration of 78% water^4,11^. Despite these limitations, it is important to emphasize that our results highlight the fact that anterior and posterior keratometry, pachymetry, and ISV index show significant circadian variations.

It’s important to know, that the light/dark cycle is important for normal corneal growth and development and the renewal of the corneal epithelium shows a daily rhythm, and also the exposition of constant light, dark, or even jetlag can modify the diurnal pattern of corneal epithelial miosis and clock gene expression^3,35^. Sleep deficiency has become a common public health problem all over the world and staying up all night could lead to tear hyperosmolarity and reduce tear secretion^22^. Sleep deprivation has many effects on health including ocular surface abnormalities. 24 h of sleep deprivation induces dry eyes with increased tear film hyperosmolarity, shortened tear break-up time, and reduced tear secretion^36^. Tears provide nutrient support and lubricate the ocular surface, and sleep deprivation results in corneal epithelial lipid accumulation, microvilli morphologic changes, and decreased tear production consequently dry eye in a mouse model^37^. Interestingly based on our study, the overnight swelling of the cornea and the diurnal changes of corneal parameters occur even without night sleep or closing the eyelids. This nocturnal fluctuation is of a greater magnitude than the daytime fluctuation, even though the participants worked actively with night light without sleeping. The reasons for this are yet unclear, and it remains an open question warranting future study. Another interesting area of research is the investigation of the effects of jet lag and its adaptation time.

## Conclusion

Our investigation utilized sleep-deprived yet healthy individuals as a cohort sample to help detect circadian fluctuations and the use of Pentacam to provide reliable and reproducible data. With a wide age range, our study stands as the first comprehensive analysis of nocturnal circadian variations, presenting robust data in comparison to preceding studies.

In conclusion, our results significantly contribute to comprehending circadian alterations in both the anterior and posterior corneal surfaces exerting an influence on the quality of vision. Notably, our study reveals that overnight swelling of the cornea and diurnal changes of corneal parameters occur even without nocturnal sleep or eyelid closure. Further research, particularly involving individuals with high refractive errors and/or individuals who have undergone refractive surgeries, is imperative to validate and elucidate the clinical implications. Recognizing diurnal variations holds pivotal importance in interpreting clinical findings, and understanding the repercussions of sleep deprivation is a research avenue that merits exploration.

## Data Availability

The datasets analyzed during the current study are available from the corresponding author upon reasonable request.
